# Surgically tunneled femoral IABP with Dacron graft: novel technique to facilitate rehabilitation before and after cardiac surgery

**DOI:** 10.1007/s12055-025-02049-9

**Published:** 2025-09-09

**Authors:** Kenny K. Nguyen, Simrat Jassal, Maryknoll Linscott, Nandini Nair, Balakrishnan Mahesh

**Affiliations:** 1 Penn State College of Medicine, Hershey, USA; 2 Heart & Vascular Institute, Penn State Health, Milton S. Hershey Medical Center, Hershey, PA USA; 3 Penn State Health Cardiac Surgery, Penn State College of Medicine, MC-H165; 500 University Drive, Hershey, PA 17033 USA

**Keywords:** Prehabilitation, Orthotopic heart transplantation, Intra-aortic balloon pump

## Abstract

We present a 52-year-old male with end-stage systolic heart failure secondary to dilated non-ischemic cardiomyopathy and recurrent ventricular tachycardia, requiring cardiac resynchronization therapy defibrillator (CRT-D) and intra-aortic balloon pump (IABP) support for cardiogenic shock. Initially supported with a percutaneous femoral IABP, the patient developed significant lower limb deconditioning. To mitigate this, a surgically placed tunneled Dacron graft IABP was introduced to improve mobility and reduce atrophy, enabling structured prehabilitation while awaiting orthotopic heart transplantation (OHT). This approach allowed for safe ambulation and physical therapy without compromising device function, ultimately enhancing functional status pre-OHT. Post-transplant, he required temporary veno-arterial extracorporeal membrane oxygenation (ECMO) and IABP support for primary graft dysfunction and was eventually weaned off devices. Another case involved a 63-year-old male transferred from an outside hospital (OSH) with 95% left mainstem stenosis and diffuse triple vessel coronary artery disease (preoperative right femoral IABP was placed). After emergency triple vessel coronary artery bypass grafting (CABG), he required left femoral veno-arterial ECMO for poor right ventricular (RV) function. He recovered postoperatively, had subsequent ECMO decannulation and IABP replacement with tunneled graft, and was able to engage in physical therapy, with improvement in upright tolerance. Further research is needed to assess infection risks and validate the promising tunneled Dacron graft technique.

## Introduction

Mechanical circulatory support devices like intra-aortic balloon pumps (IABP) remain integral in the management of advanced heart failure. Traditionally, axillary IABPs have been favored due to the associated ease with ambulation and rehabilitation. However, axillary IABP placement carries its own risks, including vascular injury and neurovascular complications, during insertion as well as during changes required due to IABP malfunction. We describe an innovative surgical technique involving a surgically tunneled Dacron graft anastomosed to the femoral artery for IABP placement. This approach preserves the accessibility of the femoral approach while enabling safe mobilization, bridging the gap between support and rehabilitation. We present two cases in which this technique was successfully implemented—one in a transplant candidate and the other post-coronary artery bypass grafting (CABG) with post-cardiotomy shock—both of whom benefited from enhanced mobility and structured physical therapy without compromising hemodynamic support. These cases highlight the potential of a graft-based femoral IABP approach to improve functional outcomes.

## Case presentation

A 52-year-old male with end-stage systolic heart failure with reduced ejection fraction (25%) secondary to dilated non-ischemic cardiomyopathy (NICM) with recurrent ventricular tachycardia requiring cardiac resynchronization therapy defibrillator (CRT-D) presented for a routine heart catheterization, which was complicated by ventricular tachycardia, necessitating implantable cardioverter defibrillator (ICD) shock and insertion of a right femoral IABP for the management of cardiogenic shock. On account of recurrent ventricular tachycardia on anti-arrhythmic medications, he was not suitable for transition to the newer micro-axial intraventricular support devices. He was listed as status 2 for orthotopic heart transplantation (OHT) due to stage D systolic heart failure requiring both inotrope (milrinone) and mechanical support. After 3 weeks of percutaneous IABP therapy, physical and occupational therapy observed deconditioning with lower limb muscle atrophy. The decision was made to transition to a surgically placed IABP to facilitate mobilization and minimize further lower limb muscle atrophy. Following the transition to a surgically placed IABP, the patient began a mobilization program while further awaiting OHT that included therapeutic exercises (range of motion, strengthening, journaling) and functional mobility (progressing to walking at least 540 feet with a rolling walker) without disturbance of the femoral IABP (limit hip flexion to < 30°; 40–45° when transitioning from bed). The patient had several such sessions following the surgical insertion of the IABP. After 3 weeks of surgical IABP placement and prehabilitation, there was marked improvement in mobility level from 3 to 5 and lower limb muscle mass prior to orthotopic heart transplantation. Following cardiac transplantation, due to primary graft dysfunction, he required perioperative left femoral veno-arterial extracorporeal membrane oxygenation (ECMO) support, in addition to IABP support. Following the recovery of cardiac function, he was weaned off IABP and ECMO support on postoperative day (POD) 4. The patient’s condition continued to improve with decreasing requirements for inotropic support and was discharged on POD 41 with course complicated by lymphedema which improved with conservative management and prophylactic antibiotics (vancomycin, fluconazole, and cefazolin) per institutional policy. His functional mobility improved to independence while gait increased from 50 to 500 feet with a walker and was able to navigate stairs.

Our second patient (63-year-old male) received emergency CABG in the presence of a right IABP, with subsequent addition of veno-arterial ECMO after weaning off bypass for post-cardiotomy cardiogenic shock. His postoperative course was uncomplicated and on POD 10, he had his ECMO decannulated and had his percutaneously placed right IABP replaced with a surgical IABP inserted through a tunneled graft, as described below, to promote mobilization/ambulation postoperatively. He was weaned off the surgical IABP support on POD 16 with improvements following physical/occupational therapy sessions in which he tolerated increasing time spent and degrees in tilt testing (55°) with upright tolerance (gait improved to 600 feet). The patient’s condition continued to improve with decreasing requirements for inotropic support and was discharged on POD 23.

## Femoral IABP with graft

**Fig. 1:**
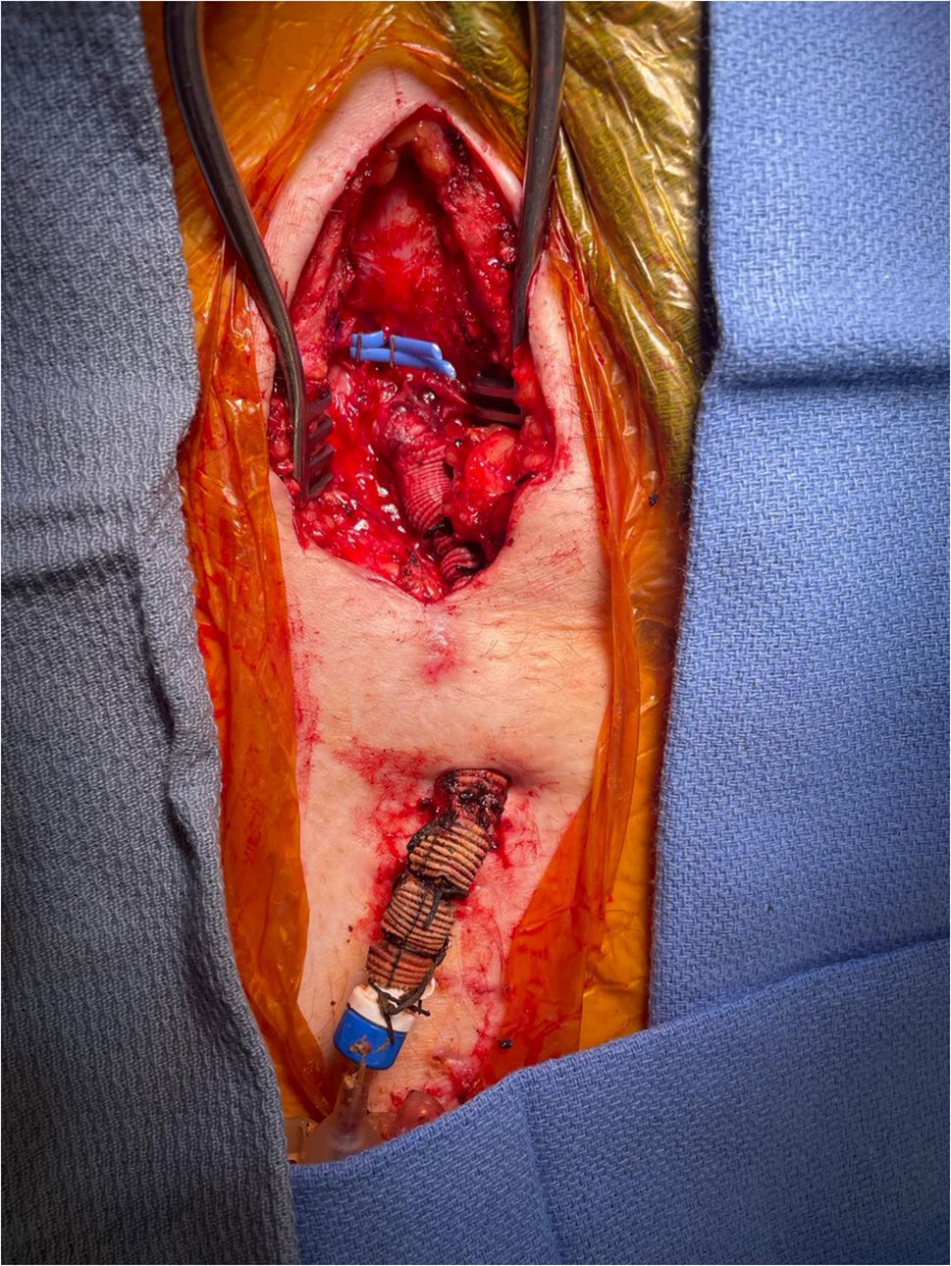
Depiction of a tunneled Dacron graft anastomosed to the right femoral artery (IABP)

**Fig. 2:**
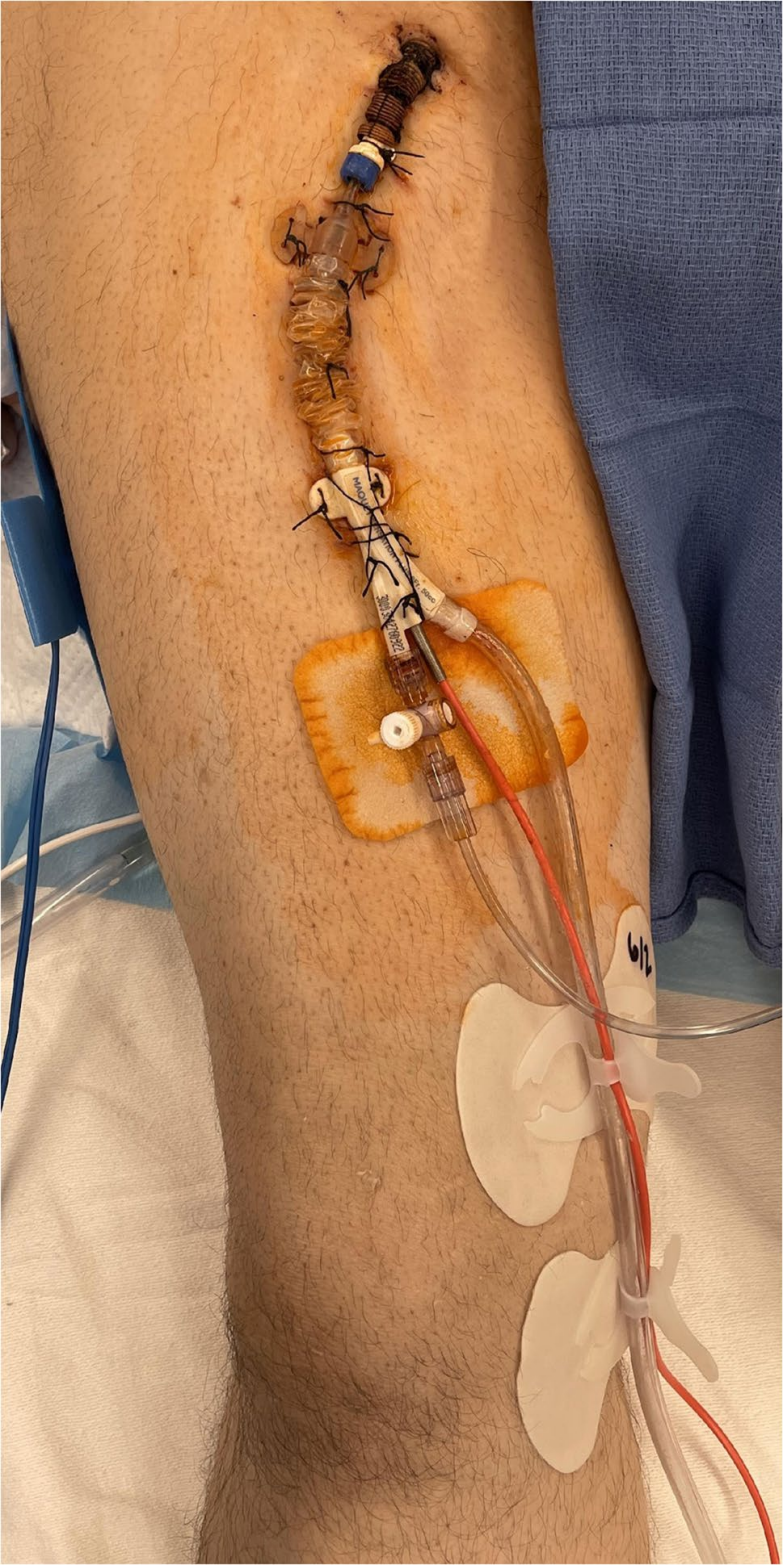
Photo of the entire setup consisting of the IABP, sheath, and tunneled Dacron graft. Legend: IABP, intra-aortic balloon pump

A longitudinal incision was made over the right femoral vessels. The right femoral artery housing the percutaneous IABP was exposed, and encircling vessel loops were placed around the artery proximally and distally. Following administration of 5000 units of heparin, the vessel loops were tightened proximally and distally, and the percutaneous IABP was explanted. A longitudinal arteriotomy was fashioned at the same site. An embolectomy was performed proximally and distally. An 8-mm Dacron graft was beveled and anastomosed end-to-side to the femoral artery using continuous 5–0 polypropylene suture. The graft was then tunneled through the skin inferior to the incision and secured with silk sutures. The sheath for the intra-aortic balloon was secured inside the Dacron graft using silk sutures, and care was taken to not advance the sheath into the femoral artery in order to minimize the potential for limb ischemia. Through this, the balloon guidewire was passed, and its position was confirmed to be in the proximal descending aorta under fluoroscopic guidance. Over the guidewire, the intra-aortic balloon was passed through the sheath and its tip was confirmed to be just distal to the left subclavian artery under fluoroscopic guidance. The guidewire was withdrawn, and the balloon channel was flushed with heparinized saline, and IABP counter-pulsation commenced at 1:1. The IABP, Dacron graft, and sheath were secured to the right lower limb using multiple silk sutures (Figs. 1 and 2). The incision was closed with absorbable polyglactin sutures in layers. In our second patient, after the original IABP was removed, an endarterectomy of a calcified plaque in the right common femoral artery was required, followed by an anastomosis with an 8-mm Dacron graft beveled to a 45° angle (end-to-side) with continuous 5–0 polypropylene suture. The remaining steps mirrored the prior technique. The patient was continued on 1:1 intra-aortic balloon counter-pulsation support and milrinone intravenous infusion. The patient was systemically heparinized for a partial thromboplastin time goal of 40 to 50 s for the duration of IABP therapy.

## Removal of the IABP

The right groin incision was reopened and the IABP was withdrawn after dividing the holding sutures. The vessel was encircled with loops proximally and distally. The Dacron graft was removed completely. The arteriotomy was repaired with bovine pericardial patch and continuous 5–0 polypropylene suture. Proximal and distal femoral embolectomies were performed with a #4 Fogarty embolectomy catheter (Edwards Lifesciences, Irvine, CA). Then, the incision was closed in a standard manner with polyglactin sutures after irrigation with copious amounts of aqueous iodine solution.

## Discussion

Since the United Network for Organ Sharing allocation policy changed in 2018, IABP utilization (bridging IABP to OHT) has increased threefold with improved waitlist outcomes and comparable post-OHT survival, thus appearing to be an effective strategy [1]. However, given the unpredictable and potentially prolonged waiting times, physical deconditioning and atrophy pose a significant risk, especially in bridge to transplantation (BTT) patients. Multimodal prehabilitation has been shown to improve short-term post-transplant outcomes due to physical status without increasing costs [2]. As such, an axillary IABP is used because it enables patients to ambulate, thereby improving mobility and conditioning [3–5]. However, despite maintaining the opportunity to rehabilitate, axillary IABPs can result in significant vascular injury and increased morbidity [6, 7]. The IABP Shock II trial showed that the risk of stroke is not significantly increased with IABPs vs. control group, while another meta-analysis showed that IABP insertion in patients with myocardial infarction increased the risk of severe bleeding requiring blood transfusion and stroke without a risk reduction of reinfarction, recurrent ischemia, or new heart failure [8, 9]. Percutaneous IABP insertion into the femoral artery via a sheath-less technique [7] has been reported, while other studies using a modified Ramsey protocol with a tilt table for early mobilization in patients with femoral percutaneous IABPs as BTT showed no significant complications associated with ambulation and helped prevent deconditioning [10, 11]. Another case series highlighted the safety for ambulation with a percutaneous femoral IABP in various age groups with increased gait speed over time [12]. In our approach, we chose a femoral IABP over the axillary approach given the ease of access of the former and the potential complications associated with the latter (increased risk of mispositioning, obstruction of subclavian/carotid arteries, pseudoaneurysm of the axillary artery, device failure due to curved vascular geometry, injury to left-sided ICD devices or to brachial plexus during left axillary artery IABP placement, and cerebrovascular accidents if right axillary IABP changes were required) [2, 7].

Although limited, prior reports have described using Dacron grafts anastomosed to the external iliac arteries or existing aortobifemoral grafts [13, 14]; those approaches typically involved complex retroperitoneal dissections or graft-to-graft anastomoses in patients with significant vascular disease. These techniques, while innovative, carry substantial risks—including retroperitoneal bleeding, infection, and complicated balloon exchanges. In contrast, our technique of directly anastomosing a Dacron graft to the common femoral artery offers a more straightforward and safer surgical alternative. It avoids retroperitoneal dissection, reduces infection risk, and allows for simple bedside IABP exchanges—all while preserving device integrity and enabling patient ambulation. This mobility, often only seen with axillary approaches, becomes possible without the associated vascular complications.

We recognize that IABP support serves a distinct role and is not a substitute for micro-axial left ventricular assist devices (LVADs) in the management of patients with profound cardiogenic shock [15]. Rather, we used this approach selectively in patients needing only modest hemodynamic support—roughly a 10–20% boost in cardiac output—to maintain end-organ perfusion in order to prevent renal or hepatic dysfunction. For patients with sensitive/unstable ventricles who are prone to intractable ventricular tachycardia, avoiding high-dose inotropes was especially important. These individuals would otherwise require aggressive anti-arrhythmic regimens that could pose serious risks to a future cardiac allograft post-transplant.

## Conclusion

The innovative integration of a femoral IABP with a tunneled Dacron graft can improve patient mobility and participation in prehabilitation without compromising the device’s functionality. This novel approach combines the benefits of the flexibility of axillary IABPs with reduced vascular complications, ultimately leading to improved patient outcomes in functional status and post-transplantation. However, further studies are needed to evaluate infection risks associated with graft-based approaches.

## Data Availability

Data are available from the corresponding author on request.
